# Inclusive Leadership Promotes Challenge-Oriented Organizational Citizenship Behavior Through the Mediation of Work Engagement and Moderation of Organizational Innovative Atmosphere

**DOI:** 10.3389/fpsyg.2020.560594

**Published:** 2020-11-27

**Authors:** Lu Chen, Fan Luo, Xiaomei Zhu, Xinjian Huang, Yanhong Liu

**Affiliations:** ^1^School of Management, Nanchang University, Nanchang, China; ^2^School of Economics and Management, East China Jiaotong University, Nanchang, China; ^3^Department of Contemporary Chinese Studies, The Chinese University of Hong Kong, Hong Kong, China; ^4^School of Management, Beijing Union University, Beijing, China; ^5^School of Economics and Management, Nanchang University, Nanchang, China

**Keywords:** inclusive leadership, challenge-oriented organizational citizenship behavior, work engagement, organizational innovative atmosphere, high-tech industries

## Abstract

Challenge-oriented organizational citizenship behavior or the organization-improving tasks employees perform beyond their job description is important for high organizational performance, but the organizational factors influencing it are poorly understood. In this study, we explored how inclusive leadership influences employees’ challenge-oriented organizational citizenship behavior in the Chinese context, drawing on data from 558 employees in high-tech industries. Multivariate correlation analysis showed that inclusive leadership promotes employees’ challenge-oriented organizational citizenship behavior and that this influence is partly mediated by work engagement. Further, it showed that organizational innovative atmosphere has a moderating effect on the relationship between inclusive leadership and employees’ challenge-oriented organizational citizenship behavior. In effect, this study expands the range of predictive variables for challenge-oriented organizational citizenship behavior and provides not only theoretical insight but also practical guidance for leaders who seek to motivate this behavior in their employees.

## Introduction

In the current rapidly developing economy, standards for organizational flexibility and innovation are constantly increasing. However, relying solely on the wisdom of managers or creating a harmonious organizational atmosphere will not effectively improve organizational performance (Yan et al., 2016), nor will it mitigate the competitive pressures and production issues faced by organizations (LePine and Van Dyne, 1998). Rather, organizations need employees to assume more responsibilities. Organizational changes and innovation needs have not only changed employees’ work content but also their work environment, increasing the proportional effect of employees’ extra-role behaviors. In view of the fact that extra-role behavior is an important factor in organizational development, the question of how to effectively stimulate employees’ extra-role behavior has become the focus of organizational behavior research (Van Dyne et al., 1995; Mackenzie et al., 2011; Wang and Chang, 2019).

Organizational citizenship behavior (OCB) is one variety of extra-role behavior, and it can be divided into affiliation-oriented organizational citizenship behavior (AOCB) and challenge-oriented organizational citizenship behavior (COCB) according to the service object and nature (Van Dyne et al., 1995). Though scholars have thoroughly investigated AOCB, COCB is just starting to attract scholarly attention due to its proven significance for organizational change and innovation (Seppälä et al., 2012; Podsakoff et al., 2014; Li et al., 2016). COCB refers to employees’ active and willful participation in organizational development and performance improvement by proposing creative ideas or instigating transformative efforts related to work methods, policies, and processes. COCB manifests in a variety of forms, including advocacy, responsibility, and forward-looking behaviors, and it often involves contestation of the *status quo* and challenges to authority. Despite this variation, all COCB is performed to advance the organization (Choi, 2007; Mackenzie et al., 2011), though it can also lead to conflict.

Given the importance of COCB to the survival and development of organizations, research has begun to explore the causes of COCB and its mechanisms. Research on COCB has mainly focused on positive behavior, which is influenced by both internal (individual) and external (organizational) factors. However, while existing studies have confirmed that COCB can be influenced by individual variables—including individual personality (LePine and Van Dyne, 2001; Miao et al., 2017); individual motivational characteristics, such as behavioral orientation, learning goals, and achievement needs (Van Dyne et al., 1995; Frese et al., 1997; Sagnak, 2016; Wang and Zheng, 2019); and general self-efficacy and self-construction (López-Domínguez et al., 2013; Yan et al., 2016, 2017), there are a few studies on leadership behavior and COCB from the perspective of leadership (López-Domínguez et al., 2013; Li et al., 2016; Sagnak, 2016; Chen and Yu, 2018; Younas et al., 2020). Existing studies have shown that leadership behavior is an important antecedent variable affecting employee COCB behavior. Some studies have proven that transactional and transformational leadership (Bettencourt, 2004; Vigoda-Gadot and Beeri, 2011; López-Domínguez et al., 2013), empowering leadership (Li et al., 2016), participative leadership (Sagnak, 2016), and shared leadership affect employees’ COCB. However, there is a lack of research on whether and how these leadership behaviors, especially inclusive leadership, affect employees. Further research is necessary in order to prove it. As inclusive leadership is a new requirement for leaders and managers to change the diversified environment (Deloitte, 2015), it is also an important management condition for the emergence of COCB (Wang and Zheng, 2019).

Inclusive leadership behaviors may contribute toward employee COCB behaviors, and inclusive leadership behaviors may have an impact on COCB through specific mediating mechanisms. As a result, employees’ COCB may involve some risk and uncertainty, and this may require individuals to take risks and invest greater efforts (Choi, 2007). This, in turn, may consume an individual’s resources and generate greater pressure (Huang et al., 2010; Yan et al., 2016). Therefore, personal resources may have an important influence on the implementation of COCB. According to the conservation of resources theory (COR), if an individual has or is supported by resources, they will face lesser pressure and will be motivated by “positive work,” which will, in turn, stimulate positive behaviors (Hobfoll, 2001a). This study believes that the COR theory is helpful in explaining the internal mechanism of the influence of inclusive leadership on employee COCB, which may be achieved by providing resources for employees. The literature has also noted that employees’ work engagement mediates between multiple leadership styles and employees’ innovative behaviors (Li, 2019). Other studies have confirmed the relationship between inclusive leadership and employees’ work engagement (Xue and Li, 2017; Fang et al., 2019). Work engagement can be considered an important internal resource (Hu et al., 2020; Nie et al., 2020), which can increase the stock of employee resources to promote COCB. Based on the above logic, work engagement is selected as a mediator variable for inclusive leadership to affect employee COCB in this study. This may play an important mediating role in the relationship between inclusive leadership and COCB. This study also attempts to explore the boundary conditions under which inclusive leadership affects COCB through work engagement. Because of organizational characteristic variables, organizational innovative atmosphere affects its perception and the assessment of a high COCB (Shalley et al., 2009). Therefore, this study treats organizational innovative atmosphere as a moderating variable for inclusive leadership to influence the individual COCB process and believes that an innovative organizational atmosphere may regulate the relationship of inclusive leadership influencing COCB through work engagement.

Unlike previous studies on the relationship between leadership behavior and employee performance, most of which used the social exchange theory, this study is based on the conservation of resources theory, assuming that employees’ work engagement is a mediating variable, and the organizational innovation atmosphere is a moderating variable in the relationship between inclusive leadership and COCB. To the best of the authors’ knowledge, this study may be the first to use empirical analysis to confirm the mediating role of work engagement between inclusive leadership and COCB, and to examine the organizational innovation atmosphere as a boundary condition for inclusive leadership to indirectly affect COCB through work engagement. It not only helps clarify the essence of the influence of inclusive leadership on COCB, but also expands the existing research perspective of COCB. In practice, it helps clarify the essence of the drive for or generation of employees’ COCB, and provides new ideas and practical guidance to stimulate or manage employees’ extra-role behaviors.

## Theory and Hypotheses

### Inclusive Leadership and Employees’ COCB

The concept of inclusiveness first appeared in education (Fuchs and Fuchs, 1994), but it expanded into organizational behavior scholarship and practice as a way to understand organizational diversity and management (Msibi, 2011; Shore et al., 2011). In earlier research, inclusive leadership was described as “leaders recogniz[ing] employee contributions and encourag[ing] employees to participate” (Nembhard and Edmondson, 2006; Hantula, 2009). Hollander (2009) put forward a more systematic definition of inclusive leadership: respecting and understanding employees, assuming responsibility, and giving feedback. Based on this definition, Carmeli et al. (2010) argued that inclusive leadership promotes “openness, accessibility and availability,” acknowledges the personal value of employees, pays attention to the way employees work, respects individual diversity, listens to employees’ differentiated needs, encourages participation, and bolsters organizational support for employees. In summary, foreign scholars identify inclusiveness as central to relational leadership.

Comparative analysis of the relevant literature reveals that inclusive leadership in the Chinese context largely resembles the definition put forward by foreign scholars, though domestic scholars interpret inclusiveness through the lens of Chinese culture. Inclusive leadership can be divided into two parts, tolerance and acceptance. “Acceptance” emphasizes the acceptance of surface differences, such as gender, age, and so on. “Tolerance” focuses on the acceptance of deep differences, such as different viewpoints, behaviors, values (Wang and Zheng, 2019), mistakes, and faults (Fang and Jin, 2014; Tang and Zhang, 2015). In the context of Chinese culture, inclusive leadership reflects the classical sentiment “the sea (inclusive leadership) embraces all rivers (employees).”

Inclusivity may be a condition of possibility for COCB, given its openness to individual differences—including different leadership styles. COCB challenges an organization’s working methods, regulations, and other conditions to improve task performance or organizational performance and may therefore lead to internal conflicts. As such, COCB relies not only on individual subjective initiative (Van Dyne et al., 1995; Frese et al., 1997; Morrison and Phelps, 1999; LePine and Van Dyne, 2001; Sagnak, 2016; Miao et al., 2017) but also on encouraging leadership style and behavior (López-Domínguez et al., 2013; Li et al., 2016; Sagnak, 2016). Indeed, research has shown that inclusive leadership enables employees’ innovative behavior (Fang et al., 2019; Zhou et al., 2018), voice (Shi and Liang, 2015; Chen and Yu, 2018; Qi et al., 2019), constructive deviant behavior (Wang and Tian, 2019), and other forms of COCB. In other words, inclusive leadership communicates to employees that their ideas and opinions about work are accepted, recognized, and respected, and that they need not worry about the negative consequences of COCB, focusing instead on the positive potential of COCB.

In view of the above analysis, this study is based on the COR theory. It explores how inclusive leadership motivates employees’ COCB behaviors. (1) Inclusive leaders can provide lots of emotional resources. Inclusive leadership may positively promote employees’ COCB in three ways. Inclusive leadership fosters emotional trust between leaders and employees based on mutual respect and understanding. Organizational citizenship behavior is an extra-role behavior, which requires employees to invest in their own cognition, emotions, energy, and other resources. When these resources are lacking, employees will have tension (McNeely and Meglino, 1994). Senior leadership is an important source of valuable resources for employees in the workplace (Demerouti et al., 2001), especially when an inclusive and respect-oriented leadership style is applied. Inclusive leadership, characterized by “openness,” “accessibility,” “understanding and recognition,” etc., often provides psychological and work support for employees experiencing difficulties (Guo et al., 2018), and encouraging employees to propose new ideas and share their visions not only helps them identify the problems and development opportunities in the current organization but also inspires them to actively implement COCB behaviors that are beneficial to organizational development (Wang and Zheng, 2019). Although employees’ hard work and challenging behaviors can lead to the loss of personal resources, the behavioral characteristic of inclusive leadership aims to establish an emotional bond between employees and leaders (Nembhard and Edmondson, 2006; Ryan, 2006; Hollander, 2009; Javed et al., 2017; Sanchez-Famoso et al., 2017; Wang and Zheng, 2019). According to the conservation of resources theory (COR), inclusive leadership behaviors complement resources that play an important role for individual employees who are more inclined to share concerns and take responsibility when their efforts are reciprocated (Wang and Zheng, 2019). (2) Inclusive leadership can provide a great deal of conditional resources. The accessibility of inclusive leaders means that employees can consult leaders at any time when they encounter problems and have equal dialogue with leaders. This facilitates two-way communication. Inclusive leadership gives employees a high degree of freedom, encouraging them to share information on an equal basis and advocating open discussion about solving problems and achieving goals. This creates a harmonious organizational atmosphere, in other words, when employees interact with inclusive leaders or other members of the organization, they can obtain more conditional resources that are similar to organizational support (Choi et al., 2015), and supplement or enhance their sense of acquisition of their own resources. Employees will do more to generate creative ideas. Employees feel empowered to take responsibility and be proactive, and make constructive COCB behavior to promote the sustainable development of the organization (Javed et al., 2017; Wang and Chang, 2017). (3) Inclusive leadership provides a wealth of cognitive resources. Inclusive leaders give employees support and help employees overcome various difficulties that they encounter in the process of implementing COCB behavior (Parker et al., 2010). When employees encounter difficulties, inclusive leaders can use their accumulated professional knowledge, skills, and experience to guide and help employees, to guide them to creatively explore and practice solutions to existing problems in the organization, and to give employees more space for personal talents (Tang et al., 2015). As COCB behavior often involves questioning and challenging the current working methods and policies of the organization, such behaviors have certain risks and encounter many difficulties (Bodankin and Tziner, 2009). Inclusive leaders give employees professional guidance and assistance and offer a wide range of cognitive resources, in order to help the staff access the resources they need in order to implement COCB behavior. Furthermore, inclusive leadership fosters this by recognizing and respecting differences, giving employees various opportunities to express their opinions and ideas, promoting face-to-face communication, expressing concern about team and organizational development, conveying support to employees, encouraging employees to innovate, and generally enhancing employees’ relationship with the organization, which reflects their respect, recognition, and help for employees, which is conducive to the establishment of high-quality exchange relations between leaders and employees (Carmeli et al., 2010). When a good relationship is formed between leaders and employees, the risk of employees pursuing innovation is obviously smaller. At this time, employees dare to engage in COCB behaviors actively. Previous studies have demonstrated inclusive leadership’s positive impact on employee motivation and engagement, showing how it promotes employee engagement in innovative work (Carmeli et al., 2010) and reduces employee silence behavior (Li et al., 2016). Based on the above analysis, we propose the following hypothesis:

Hypothesis 1: Inclusive leadership has a significant positive impact on employees’ COCB.

### Inclusive Leadership and Work Engagement

In recent years, with the rise of positive psychology research, work engagement has become a prominent topic in the field of organizational behavior and psychology. Schaufeli et al. (2002) described work engagement as a positive and fulfilling emotional and motivational state related to work, identifying three performance characteristics: vigor, dedication, and absorption. Compared to individuals with low work engagement, individuals with high work engagement usually put more psychological and physical effort into their work (Schaufeli, 2012). High work engagement means that employees dedicate more time, resources, and energy to work, and it therefore requires a leadership style that can continually and positively sustain employees’ enthusiasm for work.

Rabinowitz and Hall (1977) pointed out that leadership style is an important part of the work environment that has a significant impact on employees’ work engagement. Inclusive leadership pays attention to the establishment of good relationships with employees. At the same time, they show care and respect for employees while working with them, which should have a certain impact on such employees’ attitudes and performance. Carmeli et al. (2010) also confirmed that there is a significant relationship between inclusive leadership and employees’ work engagement and innovative behavior. Accordingly, this study speculates that inclusive leadership is beneficial for the improvement of employees’ job engagement.

According to COR theory, first, work engagement is a positive emotional–cognitive state, that is closely related to leaders. Inclusive leadership respects the differences among, and values of, employees. At the same time, it gives employees attention, care, and encouragement. This is especially important when employees are frustrated or emotionally unstable. Care and understanding from the leader can enhance the employees’ positive emotional experience, provide them with emotional resources, improve the employees’ psychological well-being, and stimulate their enthusiasm for work (Strom et al., 2014). Second, high work engagement often means that employees have to invest more time, energy, and other resources. Compared with other leaders, inclusive leaders are more tolerant toward employees and can give them more room and time for growth and development. Inclusive leaders are open-minded, willing to listen to employees’ opinions, and support employees to boldly try and make mistakes. Even when employees fail, they do not overly criticize them (Hirak et al., 2012; Peng et al., 2017). This provides positive feedback for employees.

When employees encounter work difficulties, inclusive leaders usually offer guidance and provide them with several cognitive resources to improve their work engagement. Resources are important predictors of work engagement (Bakker et al., 2014). Positive emotions that form part of the work engagement allow individuals to be flexible, explorative, and creative (Fredrickson, 2001). Research supports this, showing that work engagement explains the relationship between job resources and personal initiative (Hakanen et al., 2008), creativity (Bakker and Xanthopoulou, 2013), and innovative behavior (Park et al., 2014).

In sum, when employees perceive the support and help provided by inclusive leaders, and employees perceive these “things that help them achieve their goals” (Halbesleben et al., 2014), they will be motivated to reciprocate. They will also be more engaged in fulfilling their job roles and will be more aware of the emotional and physical resources that are invested in the organization (Strom et al., 2014). In addition, inclusive leadership could encourage employees to make greater contributions to their organizations (Hollander, 2009; Strom et al., 2014; Choi et al., 2015). As has been shown, when such leaders provide appropriate challenges and support, they have encouraged followers to exceed their job requirements (Detert and Edmondson, 2011). Moreover, inclusive leadership may improve employees’ job satisfaction by showing unique openness and accessibility to the employees. Such inclusive leadership results in job satisfaction that will ultimately have a positive effect on employees’ work engagement (Hollander, 2009; Nishii, 2013).

In a relational society like China, employees usually attach great importance to interactions with leaders. Inclusive leadership, with its unique openness and accessibility, helps to establish better communication mechanisms and emotional interaction mechanisms with employees. According to the social exchange view (Blau, 1964), when employees perceive leaders as helpful and compassionate, they will reciprocate this investment through practical actions such as hard work. Leaders’ attention and concern are especially significant when employees are frustrated, as they help to establish a more solid psychological relationship between employees and leaders, strengthening employees’ organizational commitment (Yan et al., 2017) and stimulating their enthusiasm for work. Based on the above analysis, we propose the following hypothesis:

Hypothesis 2a: Inclusive leadership has a significant positive impact on work engagement.

### Work Engagement and Employees’ COCB

Challenge-oriented organizational citizenship behavior is potentially risky because it may cause unnecessary conflicts with leaders or colleagues. However, work engagement may partially mitigate employees’ risk aversion, thereby promoting COCB. Harter et al. (2002) showed that work engagement is a positive working state in which employees associate themselves with work. Employees with high work engagement will be more tenacious when faced with difficulties, maintaining their work enthusiasm and dedication longer than employees with low work engagement. They are willing to accept challenges and still concentrate on their work, and they often experience more positive physical and mental states at work than their peers.

According to the COR theory, employees with high work engagement experience positive emotions at work (Schaufeli et al., 2006), such as happiness, joy, and enthusiasm, and accumulate positive emotional resources. They are more likely to exhibit creative and innovative behaviors (Agarwal et al., 2012). Under the COR theory, this is attributed to an individual’s investment in resources to avoid possible losses in the future (Vinokur and Schul, 2002; Ito and Brotheridge, 2003). Some empirical studies have examined the investment behavior of individuals in an organization in the context of resource loss. When engaged in work, individuals invest a lot of time, energy, and other physical and mental resources. When these resources cannot be replenished in time, a negative mental state of emotional exhaustion will follow (Hobfoll, 2001b). Halbesleben and Bowler (2007) found that when emotions are exhausted, employee performance declines, but organizational citizenship behavior increases, which is beneficial for colleagues and supervisors. This is because interpersonal relationship-oriented organizational citizenship behavior has a strong tool value for employees at this time. Employees expect to obtain resource returns from supervisors and colleagues quickly through the reciprocal mechanism of social exchange, and to avoid further loss of existing resources.

Furthermore, work engagement not only stimulates employees’ enthusiasm for work but also enhances their sense of identification with the organization. Employees who regard organizational goals as their own goals will generate more ideas and exhibit more COCB, such as questioning or challenging proposals that will lead the organization in the wrong direction. These individuals make improvements that are conducive to the development of the organization. In addition, employees with high work engagement tend to assume more responsibility and exhibit a clearer mission than those with low work engagement. Driven by this sense of purpose, employees with high work engagement are more willing to break the rules to provide valuable opinions, communicate with colleagues or leaders who contradict their own opinions, and attach great importance to the organization’s development (De Spiegelaere et al., 2014). Previous studies have confirmed that work engagement can predict employee well-being, job satisfaction, and extra-role behaviors (Halbesleben, 2010; Christian et al., 2011). Further, work engagement can positively affect employees’ workplace contributions (Zhao and Zhai, 2018), proactive behavior, innovative behavior (Salanova et al., 2011; Li G.H. et al., 2017; Li N. et al., 2017), and other manifestations of COCB. Based on the above analysis, we propose the following hypothesis:

Hypothesis 2b: Work engagement has a significant positive impact on employees’ COCB.

### Mediating Role of Work Engagement

The previous discussion used inclusive leadership as the antecedent of employees’ work engagement, while employees’ work was the antecedent of COCB behavior. This present study proposes that inclusive leadership can promote employees’ COCB through work engagement. This increase in work engagement will lead to various organizational citizenship behaviors that, in turn, will include COCB and are related to organizational outcomes (Hobfoll, 2002). In other words, work engagement as an intermediary promotes the interrelationship between inclusive leadership and employee COCB. This view can be explained by the COR theory, which is fundamentally about maximizing resources. In order to meet the needs of employees to be respected and the needs of maximizing social welfare, employees will strive to obtain, protect, and increase organizational resources.

Though individuals with high work engagement may benefit from OCBs, this extra-role behavior actually consumes individual resources (Halbesleben et al., 2009). According to the COR theory, individuals will do their utmost to acquire, protect, and maintain their existing individual and situational resources, treating losses as external threats and adjusting themselves to changes in the external environment (Duan et al., 2020). Hence, this kind of employee is more sensitive to leadership styles. When a leader’s behavior satisfies an employee’s needs in work engagement, the employee will feel that their resources are either retained or increased, and they will continue to exhibit work engagement.

The accumulation of resources is good for both employers and employees, and results in improved organizational efficiency, which promotes self-development (Islam and Tariq, 2018). For employees, this resource maximization ensures their investment in work, thereby saving them a great deal of resources needed to achieve higher goals (Gorgievski and Hobfoll, 2008).

As previous studies have confirmed, employees with high work engagement are more likely to exhibit innovative behaviors than those with low work engagement owing to a greater focus at work, have the capacity to bear more failures and frustrations, dare to accept challenges, and have the active pursuit of solutions to problems (Janssen, 2000).

Furthermore, we have seen that inclusive leadership can stimulate employees’ enthusiasm for work by promoting positive social exchange between leaders and employees (Choi et al., 2015; Peng et al., 2017; Xue and Li, 2017). This in turn helps employees to expand the scope of their attention, concentration, and cognition, thereby increasing the likelihood of COCB (Halbesleben, 2010; Christian et al., 2011; Zhao and Zhai, 2018). Therefore, it can be inferred that inclusive leadership stimulates employees’ work engagement, and employees’ work engagement inspires employees to exhibit COCB. Based on the above analysis, we propose the following hypothesis:

Hypothesis 2: Work engagement mediates inclusive leadership’s effect on employees’ COCB.

### Moderating Role of Organizational Innovative Atmosphere

The “broken windows” theory proposed by Wilson and Kelling (1982) reveals that environment has a substantial impact on individual behavior. Kurt Lewin’s field theory also explains the interaction between individual and environment. Lewin’s theory is based on his concept of field in individual psychology, denoting a person’s life space; that is, the person and their psychological environment as it exists for them. The life space of a group consists of the group and the environment as it exists for the group. Motivation in this context is usually related to group membership, personal ability, social channels, etc. Organizational climate refers to the general perception and feelings of organizational members in the organizational environment (Schneider, 1975). Existing research has confirmed that organizational climate has diverse effects on OCB, knowledge-sharing behavior, employee work engagement, and so on (Janz and Prasarnphanich, 2003; Hakanen et al., 2008). In fact, as an extra-role behavior, COCB will inevitably be affected by the organizational climate.

Organizational innovative atmosphere is the degree to which organizational members perceive an organization as hospitable to innovation—that is, the degree to which the organization encourages and supports innovation activities. Ramlall (2008) found that if organizational members perceive that the organizational environment is harmonious, innovative, and positive, then individuals are more likely to exhibit positive behavior. On the other hand, if there is no such atmosphere in the organization, the organizational climate will weaken members’ motivation to exhibit positive behavior. Therefore, a strong organizational innovative atmosphere can make employees feel that innovative ideas and opinions are effective and acceptable, that the organization recognizes, supports, and encourages this behavior, and that others will not discriminate against them for exhibiting COCB (Baer et al., 2003; Choi, 2007).

The facts mentioned in the preceding paragraph can be further explained by the COR theory, as individuals always actively maintain, protect, and construct their own resources (Yao and Li, 2014). This also accounts, at an organizational level, for a large number of studies in recent years that have been based on the crossover model and have provided new perspectives and feasible ways for understanding and exploring the interpersonal flow of resources within the framework of the COR theory (Hobfoll et al., 2018). The crossover effect describes the impact of individual stress and stress levels on those of other people in the same social environment (Bolger et al., 1989). This effect is essentially a transfer of mental state and experience between interpersonal relationships. Studies have shown that psychological resources, such as self-esteem, self-efficacy, and work engagement, can be transmitted among individuals within the same group or organization (such as colleagues, spouses, superiors, etc.), showing cross-effects (Neff et al., 2012, 2013; Gutermann et al., 2017). Within the organization, leaders who own and control more resources are obviously a highly important resource outflow for ordinary employees with relatively few resources. The open support of inclusive leaders can play a major role in the flow of resources by helping employees cope with resource depletion. An innovative atmosphere in the organization can help form an open organizational culture involving mutual aid that promotes the active flow of resources among the organization’s members.

In order to reduce resource loss and increase resource stock, employees will conduct a cognitive assessment of multiple behaviors to determine the types of behaviors that need to be cultivated or abandoned (Gu et al., 2017). Specifically, employees’ willingness to invest organizational resources in COCB depends on their assessment of the behavior. In a high-level organizational innovative atmosphere, employees will feel that their divergent opinions and transformative behaviors are supported and understood by their organization and colleagues. This sense of resource support alleviates employees’ worries about resource loss, enabling them to implement COCB. However, in a low-level organizational innovative atmosphere, employees will feel that their divergent opinions and transformative behaviors are rejected by their organization and colleagues, exacerbating their fear of resource loss and discouraging COCB. In this setting, even the positive impact of inclusive leadership on employees’ COCB will be weakened, further undermining COCB. Based on the above analysis, we propose the following hypothesis:

Hypothesis 3: Organizational innovative atmosphere plays a moderating role in the relationship between inclusive leadership and COCB.

**FIGURE 1:**
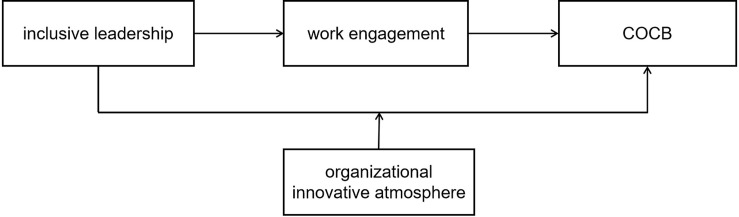
Research model.

The research model constructed in this article is illustrated in Figure 1.

## Materials and Methods

### Sample

We performed field and network research from December 2019 to March 2020, collecting data mainly from high-tech and IT industry enterprises in Jiangxi, Jiangsu, Zhejiang, Anhui, Fujian, Guangdong, and other regions. With the assistance of HR managers, we randomly selected full-time employees as participants. After receiving informed consent from participants, links to the online survey were sent to them. In order to avoid common method bias, we (1) informed the subjects before the investigation that the research results would only be used for academic research and would remain confidential; and (2) asked each enterprise to collect no more than five samples per department to prevent subjects from comparing their responses with each other. We analyzed missing values, outliers, and multicolinearity because they may affect the validity of the results. A total of 608 questionnaires were collected. The data were found to have no missing values, which may be due to the researchers’ careful attention when collecting data. After eliminating random answers (i.e., too short to complete), missing questions, and inconsistent or invalid questionnaires, 558 valid questionnaires were retained, and the effective recovery rate was 91.78%. According to the questionnaire, the ratio of men to women was close, as 295 men accounted for 52.9% of the respondents; the age group of 26–45 years accounted for the largest proportion (58.4%); employees with an undergraduate degree accounted for 48.2% of the respondents, followed by those with master’s degrees (24.6%), doctorates and post-doctorates (8.1%); the largest portion in terms of working years was 8–10 years, accounting for 26.9% of the respondents, followed by 5–7 years (20.6%); and the sample was dominated by full-time and front-line employees (74.0%).

### Research Tools

To ensure the reliability and validity of the questionnaire, we selected mature domestic and foreign measures. The organizational innovative atmosphere measure came from China, whereas the rest of the measures were derived from foreign periodicals and underwent a process of translation/back translation. To ensure that the content of each measure was not ambiguous, we recruited three English majors to assist in translation and several senior professors in the field to check the quality of the measures. All the measures used a five-point Likert scale from 1 point (completely disagree) to 5 points (completely agree).

(1) Inclusive leadership was measured with the three-dimensional, nine-item scale developed by Carmeli et al. (2010); it had an internal consistency coefficient of 0.907 in this study. (2) Work engagement was measured with the UWES-9 scale developed by Schaufeli et al. (2006); it had an internal consistency coefficient of 0.907 in this study. (3) Organizational innovative atmosphere was measured with the five-dimensional, 20-item scale developed by Liu and Shi (2009) based on the Chinese KEYS scale; it had an internal consistency coefficient of 0.955 in this study. (4) COCB was measured with the five-item scale developed by Mackenzie et al. (2011); it had an internal consistency coefficient of 0.851 in this study.

In addition, we collected demographic information as control variables, including gender, age, education, years of work experience, and position in work unit.

### Statistical Calculations

#### Confirmatory Factor Analysis (CFA)

This article used AMOS 21.0 (Chicago: IBM SPSS) to carry out the CFA, comparing the benchmark model (four-factor model) with the competition models ( one-, two-, and three-factor model). The degree of each model’s index superiority and inferiority is listed in Table 1. We can see that the fit indices for the benchmark model are obviously superior to those of the other three models (*X*^2^/*df* = 1.169, GFI = 0.925, AGFI = 0.917, CFI = 0.989, RMSEA = 0.017, IFI = 0.989, TLI = 0.988, NFI = 0.929), indicating that the four variables used in this article (inclusive leadership, organizational innovative atmosphere, work engagement, and COCB) are independent of each other and have high discriminative validity.

**TABLE 1:** Results of confirmatory factor analysis (*N* = 558).

Model	*X*^2^/*df*	GFI	AGFI	CFI	RMSEA	IFI	TLI	NFI
One-factor model	5.017	0.560	0.516	0.738	0.085	0.739	0.725	0.694
Two-factor model	4.797	0.573	0.529	0.753	0.083	0.754	0.740	0.708
Three-factor model	2.834	0.726	0.697	0.881	0.057	0.881	0.874	0.828
Four-factor model	1.169	0.925	0.917	0.989	0.017	0.989	0.988	0.929

*Four-factor model (benchmark model): inclusive leadership, organizational innovative atmosphere, work engagement, challenge-oriented organizational citizenship behavior. Three-factor model: organizational innovative atmosphere + work engagement (combined), inclusive leadership, challenge-oriented organizational citizenship behavior. Two-factor model: inclusive leadership + organizational innovative atmosphere + work engagement (combined), challenge-oriented organizational citizenship behavior. One-factor model: inclusive leadership + organizational innovative atmosphere + work engagement + challenge-oriented organizational citizenship behavior (combined).*

#### Common Method Bias

The employees surveyed used self-reporting to answer the questions and answers, and there may be common method bias. Based on this, we mainly examine from three aspects. First, in the questionnaire design process, authoritative experts and doctoral students in the field were invited to modify the questionnaire structure and measurement items. Second, during the questionnaire filling process, the order of the variables in the questionnaire was randomly arranged to ensure that that the respondent fills out the questionnaire anonymously (Podsakoff et al., 2003). Finally, in the data analysis stage, to avoid the influence of common method bias on the research, we applied a latent variable test to incorporate common method bias as a potential variable in the model. The results (Table 2) showed that *X*^2^ changed significantly after the control (Δ*X*^2^ = 146.425, Δdf = 36, *p* < 0.01). Because *X*^2^ is sensitive to sample size, other fit indices were also needed (Wen et al., 2004). The lack of variance observed in the RMSEA, CFI, and TLI fit indices was significant (*p* ≤ 0.001), indicating that there was no serious common method bias in this study.

**TABLE 2:** Variance in fit indices before and after model control.

	X^2^	df	X^2^/df	GFI	AGFI	CFI	RMSEA	IFI	TLI	NFI
Before control	1,350.101	858	1.574	0.914	0.905	0.963	0.032	0.963	0.961	0.904
After control	1,203.676	822	1.464	0.919	0.906	0.971	0.029	0.971	0.968	0.915

#### Data Aggregation Test

This study’s questionnaire was filled out by employees, but inclusive leadership and organizational innovative atmosphere can be regarded as organizational variables. As such, we had to aggregate the data before performing in-depth analysis. The results showed that the data on inclusive leadership [*r*_*wg*_ = 0.84, ICC(1) = 0.547, ICC(2) = 0.916] and organizational innovative atmosphere [*r*_*wg*_ = 0.93, ICC(1) = 0.527, ICC(2) = 0.957] met the requirements for aggregation to the organizational level.

## Results

### Descriptive Statistics and Correlation Analysis

The mean, standard deviation, and correlation among the study variables are shown in Table 3. There was a significant positive correlation between inclusive leadership and employees’ work engagement (*r* = 0.466, *p* < 0.01), inclusive leadership and employees’ COCB (*r* = 0.554, *p* < 0.01), inclusive leadership and organizational innovative atmosphere (*r* = 0.507, *p* < 0.01), employees’ work engagement and organizational innovative atmosphere (*r* = 0.569, *p* < 0.01), employees’ work engagement and COCB (*r* = 0.544, *p* < 0.01), and organizational innovative atmosphere and employees’ COCB (*r* = 0.593, *p* < 0.01). These findings preliminarily verified our research hypotheses.

**TABLE 3:** Results of correlation analysis (*N* = 558).

	Mean	Standard deviation	*N*	1	2	3	4
(1) Inclusive leadership	3.64	0.87	558	1			
(2) Organizational innovative atmosphere	3.64	0.82	558	0.507**	1		
(3) Work engagement	3.63	0.86	558	0.466**	0.569**	1	
(4) Challenge-oriented organizational citizenship	3.84	0.69	558	0.554**	0.593**	0.544**	1

*Significant (bilateral) correlation at *p* ≤ 0.05 level. **Significant (bilateral) correlation at *p* ≤ 0.01 level. ***Significant (bilateral) correlation at *p* ≤ 0.001 level.

In order to ensure the reliability of the experimental results, it is necessary to diagnose the multicolinearity of the dependent variable before performing multiple regression. The results of the multicolinearity test show that the tolerance of each independent variable is much greater than 0.2, and the variance expansion factor is much less than 5. These results indicate that there is no multicolinearity between the independent variables, and these independent variables can be incorporated into the multiple regression model.

A CFA was executed on all constructs to analyze the internal consistency, and convergent and discriminant validity (Table 4). As seen in Table 4, composite reliability (CR) ranged from 0.88 to 0.97 for each factor. These values are greater than the recommended cutoff point of 0.60 and thus confirmed the presence of inner consistency reliability among each construct (Fornell and Larcker, 1981; Bagozzi and Yi, 1988). The values of Cronbach’s α were also above 0.70 (Nunnally and Bernstein, 1994). According to Hair et al. (2010), factor loadings above 0.5 are considered significant for providing convergent validity. In our study, the standardized factor loadings ranged from 0.60 to 0.88 (*p* < 0.001). Therefore, the measures did not have any issue regarding convergent validity. To check discriminant validity, AVE estimates were compared with the squared values of correlation between the constructs. As shown in Table 3, all AVE values were greater than the squared correlations, thus the model fit the criteria for discriminant validity (Shaffer et al., 2016).

**TABLE 4:** Measurement model for all four factors.

Construct	Loading	Mean	SE	α	AVE	CR
**Inclusive Leadership**				0.92	0.63	0.91
The manager is open to hearing new ideas	0.73	3.62	1.14			
The manager is attentive to new opportunities to improve work processes	0.76	3.61	1.11			
The manager is open to discuss the desired goals and new ways to achieve them	0.76	3.66	1.11			
The manager is available for consultation on problems	0.68	3.67	1.12			
The manager is an ongoing ‘presence’ in this team-someone who is readily available	0.82	3.62	1.12			
The manager is available for professional questions I would like to consult with him/her	0.60	3.61	1.08			
The manager is ready to listen to my requests	0.68	3.64	1.11			
The manager encourages me to access him/her on emerging issues	0.71	3.66	1.15			
The manager is accessible for discussing emerging problems	0.76	3.64	1.15			
**Organizational Innovation Climate**				0.96	0.61	0.97
During work, my colleagues support and assist each other	0.83	3.72	1.13			
During work, my colleagues are willing to share each others’ methods and techniques	0.83	3.64	1.12			
My colleagues often communicate and discuss issues at work	0.75	3.66	1.14			
When I have a new idea, my colleagues actively express their suggestions and opinions	0.80	3.61	1.09			
My supervisor respects and tolerates different opinions and objections from employees	0.82	3.60	1.09			
My supervisor encourages subordinates to make proposals to improve production or service	0.78	3.65	1.10			
My supervisor will support and assist subordinates to realize their work creativity	0.71	3.59	1.13			
My supervisor is a good model of innovation	0.72	3.64	1.12			
The company advocates new attempts and learns from mistakes	0.70	3.59	1.10			
The company appreciates and recognizes innovative and enterprising employees	0.77	3.63	1.13			
The company usually rewards employees for innovative ideas	0.77	3.70	1.07			
The company advocates freedom, openness and innovation and change	0.81	3.68	1.13			
I have free time to develop ideas or find new methods	0.81	3.65	1.10			
I can obtain equipment and equipment to verify new ideas	0.76	3.56	1.10			
I can obtain sufficient information and materials for creative work	0.79	3.66	1.14			
I have plenty of time to realize my new ideas	0.78	3.65	1.08			
At work, I can complete tasks in the way I like	0.81	3.65	1.11			
My work is very challenging	0.77	3.63	1.05			
I can decide most things at work by myself	0.75	3.61	1.10			
Work arrangement can give full play to my ingenuity	0.77	3.68	1.08			
**Work Engagement**				0.91	0.70	0.95
At my work, I feel bursting with energy	0.83	3.63	1.14			
At my job, I feel strong and vigorous	0.88	3.63	1.12			
I am enthusiastic about my job	0.85	3.64	1.12			
My job inspires me	0.79	3.64	1.12			
When I get up in the morning, I feel like going to work	0.82	3.65	1.14			
I feel happy when I am working intensely	0.80	3.65	1.10			
I am proud of the work that I do	0.83	3.63	1.11			
I am immersed in my work	0.86	3.65	1.10			
I get carried away when I am working	0.84	3.58	1.12			
**COCB**				0.87	0.61	0.88
Communicate their opinions about work issues to others in the group even if their opinion is different and the others in the work group disagree with them	0.76	3.85	0.97			
Are willing to risk disapproval in order to express their belief about what’s best for the organization	0.78	3.79	0.98			
Do not hesitate to challenge the opinions of others that they feel are directing the store/company in the wrong direction	0.76	3.81	0.95			
Often try to recommend changes in organizational rules or policies that are nonproductive or counterproductive	0.78	3.91	0.96			
Are willing to voice their concerns about the direction of the work team or company	0.79	3.86	0.95			

### Hypotheses Testing

To further test our hypotheses, we performed hierarchical regressions. The results are shown in Table 5. Model 3 tested *Hypothesis 1*, and the result shows that inclusive leadership has a significant positive effect on employees’ COCB (β = 0.431, *p* < 0.05). Thus, *Hypothesis 1* was verified. Model 2 tested *Hypothesis 2a*, and the result shows that inclusive leadership has a significant positive effect on work engagement (β = 0.463, *p* < 0.05). Thus, *Hypothesis 2a* was verified. Model 4 tested *Hypothesis 2b*, and the result shows that work engagement has a significant positive effect on employees’ COCB (β = 0.428, *p* < 0.05). Thus, *Hypothesis 2b* was verified. Model 5 initially examined the mediating effect of work engagement on employees’ COCB. The result shows that, when inclusive leadership and work engagement enter the model at the same time, the influence of inclusive leadership on employees’ COCB is reduced (β = 0.298, *p* < 0.05), and work engagement has a significant positive effect on COCB (β = 0.289, *p* < 0.05). Thus, *Hypothesis 2* was verified.

**TABLE 5:** Results of regression analysis and hypothesis test.

Variables	Work engagement		Employees’ COCB			
	Model 1	Model 2	Model 3	Model 4	Model 5	Model 6
Gender	0.088	0.102	0.120*	0.069*	0.090*	0.080*
Age	0.075*	0.050*	0.059*	0.050*	0.044*	0.050*
Level of education	0.061*	0.065*	−0.006*	−0.036*	−0.025*	−0.021*
Work years	−0.069*	−0.022*	−0.070*	−0.085*	−0.064*	−0.067*
Position in the work unit	0.039*	0.035*	0.079*	0.067*	0.069*	0.050*
Inclusive leadership	−	0.463*	0.431*	−	0.298*	0.248*
Work engagement	−	−	−	0.428*	0.289*	−
Organizational innovative atmosphere	−	−	−	−	−	0.310*
Inclusive leadership × organizational innovative atmosphere	−	−	−	−	−	−0.199*
*R*^2^	0.016	0.229	0.338	0.331	0.437	0.512
Δ*R*^2^	0.007	0.221	0.331	0.324	0.430	0.505
*F*	1.709*	27.309***	46.917***	45.411***	60.930***	72.000***

*Significant (bilateral) correlation at *p* ≤ 0.05 level. ***Significant (bilateral) correlation at *p* ≤ 0.001 level.

To further ensure the reliability of the research results and further examine the mediating effect of work engagement, we used the “Bootstrap” method, setting the sampling number to 5000. The results of the test are shown in Table 6. The indirect effect of inclusive leadership on employees’ COCB (through work engagement’s mediation) was 95% CI (0.1201, 0.2270), and the interval did not contain zero. The effect value was 0.1683, and the indirect effect was significant. This indicates that work engagement plays an intermediary role between inclusive leadership and employees’ COCB. Thus, *Hypothesis 2* was further verified.

**TABLE 6:** Decomposition table of total, direct, and intermediary effects.

	Effect value	BootSE	BootLLCI	BootULCI
Total effect	0.4313	0.0281	0.3762	0.4864
Direct effects	0.2630	0.0293	0.2053	0.3206
Indirect effects	0.1683	0.0271	0.1201	0.2270

*CI, confidence interval; LL, lower limit; SE, standard error; UL, upper limit. *N* = 558. Bootstrap size = 5,000.*

We constructed an interaction item between inclusive leadership and organizational innovative atmosphere to test the moderating effect of organizational innovative atmosphere. According to Table 5, Model 6 shows that the interaction item had a significant moderating effect on employees’ COCB (β = −0.199, *p* < 0.05) after entering the interaction field, indicating that organizational innovative atmosphere plays a negative moderating role between inclusive leadership and employees’ COCB. Thus, *Hypothesis 3* was verified.

**FIGURE 2:**
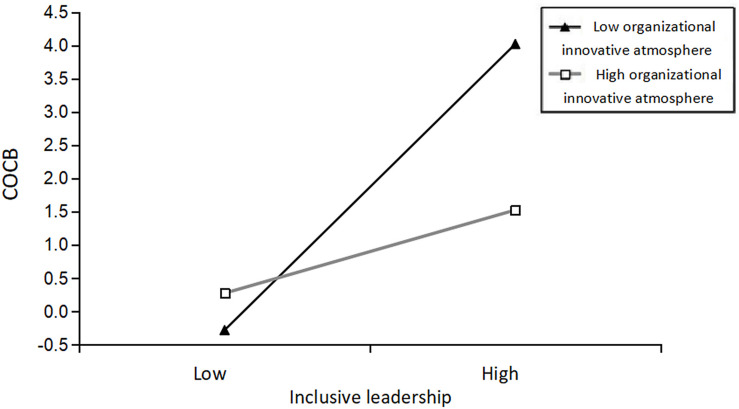
The moderating role of organizational innovative atmosphere between inclusive leadership and employees’ challenge-oriented organizational citizenship behavior (COCB).

To provide a more nuanced description of organizational innovative atmosphere’s moderating role between inclusive leadership and employees’ COCB, we used a simple slope analysis to draw a regulation effect diagram, as shown in Figure 2. We can see that the regression slope of inclusive leadership relative to employees’ COCB is positive in both low and high organizational innovative atmospheres. Organizational innovative atmosphere moderates inclusive leadership’s influence on employees’ COCB. In other words, low-level inclusive leadership may have a negative effect on employees’ COCB, but high-level organizational innovative atmosphere can mitigate this negative effect. The regression slope for a low-level organizational innovative atmosphere is slightly higher than that for a high-level organizational innovative atmosphere, indicating that the positive effect of high-level inclusive leadership on employees’ COCB is more significant in a low-level organizational innovative atmosphere than in a high-level organizational innovative atmosphere. Thus, *Hypothesis 3* was further verified.

## Discussion

The complexity and intense competition of the external economic environment forces organizations to be innovative and to change in order to survive (Tosheva and Panovski, 2014; Du et al., 2015). To meet this demand, organizations require employees’ creative ideas and behaviors in improving efficiency (Anastasiu et al., 2020; Kang et al., 2020). These transformative efforts and innovative behaviors have aroused special attention in domestic and foreign academic circles in recent years and constitute an emerging field. Consequent research has proven that leadership style, especially inclusive leadership, significantly affects employees’ OCB and innovation performance (Sui et al., 2012; Yang C.J. et al., 2020; Yang J. et al., 2020). The traits of inclusive leadership are in line with the leadership connotations of “inclusion leads to great virtue,” which are promoted in Chinese culture. By incorporating the COR theory into our research, we have shown that inclusive leadership has a positive impact on employees’ COCB partly through the mediation of employees’ work engagement, while organizational innovative atmosphere moderates inclusive leadership’s impact on employees’ COCB.

### Implications for Research and Practice

This article makes the following theoretical contributions:

First, based on the COR theory, it presents employees’ work engagement as a mechanism mediating inclusive leadership’s positive impact on employees’ COCB, thereby providing context for previous studies that have shown that inclusive leadership has a very important impact on team innovative behavior. In other words, inclusive leadership’s tolerance for individual differences and mistakes creates an open and autonomous organizational environment that encourages employees to actively participate, solve problems, and achieve goals (Hollander, 2009; Tang and Zhang, 2015; Chen and Yu, 2018; Fang et al., 2019). Under this type of leadership, employees are likely to increase their work engagement in order to maintain their existing resources, engender self-motivation and a sense of responsibility, and regard organizational goals as personal goals. Furthermore, inclusive leadership can provide continuous positive energy for COCB (Javed et al., 2019; Wang and Zheng, 2019). Prior to this study, there was no research discussion about work engagement’s mediating role between inclusive leadership and employees’ COCB, so this article can be regarded as a useful supplement to previous research.
Second, this article confirms that organizational innovative atmosphere moderates inclusive leadership’s influence on employees’ COCB. Specifically, in a high-level organizational innovative atmosphere, employees feel that their divergent opinions and transformative behavior are supported and understood by their organizations and colleagues (Ojedokun, 2012; Luo et al., 2018; Su and Xu, 2020). This support helps to relieve the pressure of resource loss and provides necessary resources for the implementation of COCB, thus enhancing inclusive leadership’s influence on employees’ COCB. In a low-level organizational innovative atmosphere, employees feel antipathy from organizations and colleagues in response to their divergent opinions or transformative behavior. In this atmosphere, employees are anxious and risk averse due to the possible loss of resources (Chen et al., 2020), and are therefore hesitant to implement COCB. Meanwhile, inclusive leadership is expected to show a higher level of performance to compensate for the impact of the low-level organizational innovative atmosphere on employees’ COCB (Jiang and Qi, 2018). These results support the view that a high-level organizational innovative atmosphere can change employees’ perception of resource loss (Li et al., 2020; Zhang and Qing, 2020). Organizations should understand that relationships between members or between members and leaders are not just transactional; they also involve commitment, responsibility, psychological cognition, and interaction (Marshall, 2005; Xu et al., 2018). We found no previous research on organizational innovative atmosphere’s moderating role between inclusive leadership and employees’ COCB. Therefore, this article meaningfully extends previous studies.
Third, this article elaborates inclusive leadership’s outcome variables. Previous studies have focused on the “acceptance” aspect of inclusive leadership, overlooking the “tolerance” aspect (Tang et al., 2015). Our results show that “mistake-tolerance” enables employees to perceive that creative ideas aimed at improving organizational performance and “high-risk,” transformative OCB are acceptable (Younas et al., 2020). This insight deepens our understanding of inclusive leadership and its effectiveness.

Moreover, this article provides the following practical guidance for organizations:

First, organizations should attempt to implement inclusive leadership because it has a direct positive impact on employee performance and OCB (Gu et al., 2017; Wang and Zheng, 2019; Younas et al., 2020). The new generation of employees pays more attention to fairness and equality, so too much emphasis on command and obedience will seriously undermine employees’ self-confidence, reducing self-efficacy, optimism, and hope while increasing emotional exhaustion and employee silent behavior (Yang et al., 2018; Zhang et al., 2018; Yan et al., 2019). This inevitably hinders organizational performance because negative mindsets are not conducive to information transmission, sharing, or efficient problem solving (Abele-Brehm, 1992; Li et al., 2013; Zhou and Mao, 2020). Leaders should gradually accept those leadership styles that promote organizational and personal growth in order to stimulate employees’ extra-role behavior and enhance overall organizational performance.
Second, managers should promote mutuality rather than hierarchy by strengthening relationships with employees, as this fosters greater work engagement. This article shows that inclusive leadership can have an indirect positive impact on employees’ COCB through the mediating role of work engagement. Thus, by actively communicating with employees and enhancing emotional investment and cognitive trust, leaders can help employees cultivate positive emotions, share constructive advice, and exhibit responsible behavior (Chen and Yu, 2018; Javed et al., 2019; Qi et al., 2019).
Third, managers should approach organizational climate as a practical intervention for work-related affairs, rather than as a superficial construct. This article shows that organizational innovative atmosphere can moderate the influence of inclusive leadership on employees’ COCB. Consequently, organizing social activities such as charity events can improve employees’ recognition of organizational culture and ideas, increase employees’ sense of responsibility, and integrate personal and organizational goals, motivating employees to protect the interests of the organization (Bashir et al., 2012; Kumari, 2014; Wang and Zheng, 2019; Yang et al., 2019; Zhong et al., 2019). Moreover, organizations can hold regular interdepartmental and even cross-departmental fellowship events to encourage communication among colleagues, inspire new ideas, improve employees’ interpersonal network, and enhance their perception of organizational support. Within this atmosphere, employees could abandon their worries about interpersonal risks and pursue COCB without anxiety.

### Limitations

First, the questionnaires in this study were all answered by employees. Future research can use matching data, for example, where employees evaluate inclusive leadership and organizational innovation atmosphere, and leaders evaluate employees’ work engagement and COCB behavior in order to minimize the common method bias. At the same time, longitudinal data at multiple time points or qualitative comparative analysis (QCA) can be used to accurately infer the causal relationship between variables. Based on the nested relationship between variables, software such as MPLUS can also be used in the future to analyze the cross-level influence mechanism of inclusive leadership on employees’ COCB.

Second, this article explored how inclusive leadership impacts individual employees’ COCB. However, inclusive leadership and organizational innovative atmosphere may also affect COCB at the team and departmental levels. In future research, cross-level paired sample studies can be used to more accurately examine the relationship between leadership style, organizational factors, and employee behavior.

Third, this article focused on the mediating role of employees’ work engagement between inclusive leadership and employees’ COCB, but other factors such as job prosperity and job well-being may reveal different influence mechanisms.

Finally, this article focused on the moderating role of organizational innovative atmosphere between inclusive leadership and employees’ COCB, but leadership style’s influence on employee behavior may also be moderated by employees’ individual characteristics, such as prospective personality, active personality, risk-taking trait, and so on. Future research should consider all of these moderating variables to deepen our understanding of employees’ COCB.

## Data Availability Statement

The raw data supporting the conclusions of this article will be made available by the authors, without undue reservation.

## Ethics Statement

The studies involving human participants were reviewed and approved by the studies involving human participants were reviewed and approved by the Ethics Committee (HREC) of the School of Economics and Management in East China Jiaotong University. The patients/participants provided their written informed consent to participate in this study.

## Author Contributions

LC and FL conceived and designed the research and methodology. LC provided guidance throughout the entire research process. LC and FL collected and compiled all of the data and literature. FL completed the calculation and analyzed the results. LC put forward the policy recommendations. XZ had revised the manuscript critically for important intellectual content. YL supplemented the English manuscript. XH revised and approved the manuscript. All the authors contributed to the article and approved the submitted version.

## Conflict of Interest

The authors declare that the research was conducted in the absence of any commercial or financial relationships that could be construed as a potential conflict of interest.
